# Design and Verification of Heading and Velocity Coupled Nonlinear Controller for Unmanned Surface Vehicle

**DOI:** 10.3390/s18103427

**Published:** 2018-10-12

**Authors:** Jiucai Jin, Jie Zhang, Deqing Liu

**Affiliations:** The First Institute of Oceanography, State Oceanic Administration, Qingdao 266061, China; zhangjie@fio.org.cn (J.Z.); liudeqing@fio.org.cn (D.L.)

**Keywords:** Unmanned Surface Vehicle, heading control, velocity control, underactuated vehicle, backstepping technology

## Abstract

Unmanned Surface Vehicle (USV) is a novel multifunctional platform for ocean observation, and its heading and velocity control are essential and important for autonomous operation. A coupled heading and velocity controller is designed using backstepping technology for an USV called ‘USBV’ (Unmanned Surface Bathymetry Vehicle). The USBV is an underactuated catamaran, where the heading and velocity are controlled together by two thrusters at the stern. The three degrees-of-freedom equations are used for USBV’s modeling, which is identified using experiment data. The identified model, with two inputs, induces heading and velocity tracking, which are coupled. Based on the model, a nonlinear controller for heading and velocity are acquired using backstepping technology. The stability of the controller is proved by Lyapunov theory under some assumptions. The verification is presented by lake and sea experiments.

## 1. Introduction

USV (Unmanned Surface Vehicle) is a novel kind of multifunctional surface platforms, which has recently received considerable attention from the control communities and oceanic application departments [1,2,3]. In recent years, different USVs are fabricated by dozens of universities and research institutes of ocean technology around the world, such as MIT and Woods Hole Oceanographic Institution, which have been applied in many oceanic fields, such as bathymetry [4], environment monitoring [5], underwater acoustics [6], marine rescue [7], and goal tracking [8,9]. Though related technologies for USVs are gradually mature, there are many challenges for USVs’ control, such as appropriate model in complex environment, stable controller, and obstacle avoidance.

The models of USVs are usually adopted as three DOF (Degrees of Freedom) equations in horizontal or four DOF equations by adding the roll motion [1,10,11], which are similar to modeling for conventional large crafts. However, due to hull type, displacement, and operation manner’s diversity, there are some differences between large crafts and small USVs’ motion model and control. System-based models are often used for large craft modeling, which are usually expressed by three Taylor series [12,13]. According to general hydrodynamic theory for marine vehicles, the quadratic form is usually used in the damping terms for the immerged vehicles, and linear form is often adopted in the damping terms for big crafts. Many works have been published for tracking control of big crafts with linear model. Based on a linear model of marine craft, a controller for path following is derived using line-of-sight algorithm and backstepping technology by Fossen [14]. A nonlinear controller for path following is designed based on a ship model with linear drag terms, but velocity control has not been considered [15]. Fossen in Reference [16] presents a nonlinear adaptive path-following controller that compensates for drift forces through vehicle sideslip, where velocity control is neglected. The path following control for an underactuated ship is proposed by passivity approach and combined cascade-backstepping approach [17]. A nonlinear adaptive path-following controller for marine craft based on a line-of-sight guidance principle is designed, where heading control is independent of velocity control [18]. In most papers for big crafts’ control, there exist some common issues or assumptions and these are not adapted for USVs: (1) the heading controllers are designed based on the linear model, while the damping terms of USVs in low velocity are different from that of big ships due to many sensors fixed outside of USVs; (2) the heading control and velocity control are designed independently thanks to rudder for big crafts or moment for control input, while double thrusters are usually used for most slow USVs, such as the USBV, and the two controllers are coupling; and (3) the key factor is that most of the designed controllers haven’t been validated in big ship experiments due to expensive cost, with the exception of using some small model ships without any disturbance in lab experiments [14,15], and thus, the usability for the controllers have not been tested well for USVs.

In recent years, since the USVs have rapidly grown, several tracking control for USVs has emerged, such as velocity, heading, path, and trajectory control. A PID heading controller of ‘Charlie’ USV is designed based on a self-oscillation identification model where velocity control is not considered [19]. Based on linear state space equations, the tracking controller design for ‘DELFIM’ USV is formulated as a discrete time H_2_ control problem and is solved using Linear Matrix Inequalities [20]. Based on the heading model for ‘Springer’ USV using system identification technology, fuzzy linear quadratic Gaussian controller has been designed and tested [21]. A Nomoto steering model is used for heading control based on a cascaded PD controller, where the nonlinear drag terms are simplified to a linear model under steady conditions [22]. Heading and velocity controllers are usually designed separately [23], or a heading controller is designed without velocity control [24,25], because complexity drastically increases if heading and velocity are stabilized simultaneously [16,26]. In the above papers, the linear drag terms are adopted in USV modeling, and the heading and velocity controls are uncoupled. However, the drag terms are not linear in actual situation due to irregular forms of USVs. In the USVs with double thrust, the heading control is achieved by regulating speeds of double thrusters, and thus, the velocity of USV will be changed during the heading control, i.e., the heading control and velocity control are coupled. 

Motivated by these recent developments in the tracking control of big crafts and USVs, this paper presents a coupled heading and velocity controller design for a realistic quadratic damping model for the USV, and the verification has been finished by lake and sea experiments. The organization of the paper is as follows: In Section 2, the quadratic model for USV is identified by experiment data. The coupled heading and velocity controller is designed using backstepping technology, which is described in Section 3. Section 4 and Section 5 present the simulation and experiment results, respectively. Finally, Section 6 concludes the paper.

## 2. The Model of the USBV

The model is the basis of the heading and velocity’s control for the USBV. In the section, the model, the hydrodynamic coefficients’ identification and thrust identifications are given out using experiment data.

### 2.1. Three Degrees-of-Freedom Equations

In the ships’ autonomous control, the three Degrees-of-Freedom equations are usually adopted to describe their horizontal motion. In the motion model, the linear form is mostly used in the drag terms. However, the USBV’s drag terms accord with quadratic form, which is validated in tow experiments, so USBV motion model is expressed as follows,

(1) $$M\dot{\nu }+C(\nu )\nu +D(\nu )\nu |\nu |=\tau$$

where *M* represents the inertia matrix, ***C*** represents the Coriolis and centripetal matrix, ***D*** represents hydrodynamic drag matrix, and ***v*** represents the linear and angular velocity vectors in horizontal, and ***τ*** represents the driven force and moment of thrusters. The above hydrodynamic matrixs are given as follows,

$$M=\left[\begin{array}{ccc} m_{11} & 0 & 0\\ 0 & m_{22} & 0\\ 0 & 0 & m_{33} \end{array}\right]=\left[\begin{array}{ccc} m-X_{\dot{u}} & 0 & 0\\ 0 & m-Y_{\dot{v}} & 0\\ 0 & 0 & I-N_{\dot{r}} \end{array}\right]$$

$$C(\nu )==\left[\begin{array}{ccc} 0 & 0 & -m_{22}v\\ 0 & 0 & m_{11}u\\ m_{22}v & -m_{11}u & 0 \end{array}\right]$$

$$D(\nu )=\left[\begin{array}{ccc} d_{11} & 0 & 0\\ 0 & d_{22} & 0\\ 0 & 0 & d_{33} \end{array}\right]=\left[\begin{array}{ccc} X_{u|u|} & 0 & 0\\ 0 & Y_{v|v|} & 0\\ 0 & 0 & N_{r|r|} \end{array}\right]$$

**Figure 1:**
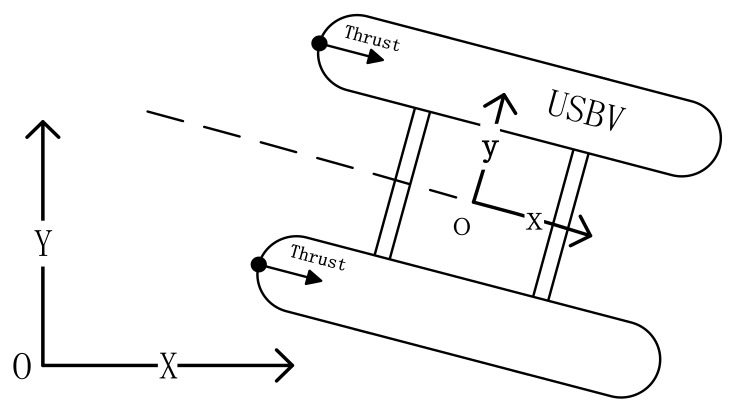
Earth-fixed coordinate axes O-XY and body-fixed coordinate axes o-xy.

In the body-fixed coordinate axes o-xy and the earth-fixed coordinate axes O-XY (Figure 1), the transformation relationships between positions and velocities are as follows,

(2) $$\begin{array}{l} \dot{x}=u\cos\psi -v\sin\psi \\ \dot{y}=u\sin\psi +v\cos\psi \\ \dot{\psi }=r \end{array}$$

According Equation (1), the model of the USBV can be described by

(3) $$\begin{array}{l} \dot{u}=\frac{m_{22}}{m_{11}}vr-\frac{d_{11}}{m_{11}}u\left|u\right|+\frac{1}{m_{11}}\tau_{1}\\ \dot{v}=-\frac{m_{11}}{m_{22}}ur-\frac{d_{22}}{m_{22}}v\left|v\right|+\frac{1}{m_{22}}\tau_{2}\\ \dot{r}=\frac{m_{11}-m_{22}}{m_{33}}uv-\frac{d_{33}}{m_{33}}r\left|r\right|+\frac{1}{m_{33}}\tau_{3} \end{array}$$

Thus, Equations (2) and (3) are composed as the model of the USBV, where [*x*, *y*, *ψ*] represent the position and orientation in the earth-fixed coordinate axes, and [*u*, *v*, *r*] represent the surge velocity, sway velocity, and yaw angular velocities, respectively, in the body-fixed coordinate axes, and [*m*_11_, *m*_22_, *m*_33_] represent inertia mass, and [*d*_11_, *d*_22_, *d*_33_] represent drag coefficients, and *τ*_1_, *τ*_2_ represent thrusts in x-axis and y-axis respectively, and *τ*_3_ represents thrust moment. It is noted that the value of *τ*_2_ equals to zero because there is not a propeller or a rudder for USBV in the y axis.

### 2.2. Identification for Drag Coefficients

In most system identification for ships, coefficients of the motion model are entirely identified using experiment data. However, the way for whole coefficient identification will need large and complete experiment data, which is expensive and complicated. Thus, the part coefficient identification is usually carried out in USV modeling. In small catamaran USV modeling, the drag coefficients are important hydrodynamic parameters, which are dominant in USV maneuvering and endurance ability.

The drag coefficients *d*_11_, *d*_22_ and *d*_33_ in drag coefficient matrix ***D*** will be acquired using USBV’s stable state data in experiments. When the USBV’s line motion is stable, i.e., $\dot{u}=v=r=0$, and $\tau_{1}-d_{11}\cdot u\left|u\right|=0$. USBV towed experiments are executed using an ergometer at a lake in November 2013 in Qingdao, China. The coefficient *d*_11_ is obtained using a quadratic equation regression. When difference of revolving speed between left and right thrusters is fixed and their rotation direction is same, USBV will form stable circle motion, thus $(X_{\dot{u}}-m)ur-d_{22}v\left|v\right|=0$. Thus, the coefficient *d*_22_ is also calculated based on the experiment data in 2013, lake tested using a quadratic equation’s regression. When the left and right thrusters’ revolving direction is converse and the revolving speed is the same, USBV will form a stable rotation motion around a fixed point, and thus we can get *d*_33_ using a quadratic equation’s regression. The remainder coefficients in Equation (3) are calculated based on the theoretical method [10], and all of hydrodynamic coefficients are listed in Table 1.

### 2.3. Thrust and Moment

In the USBV’s dynamic system, two brushless DC thrusters are fixed in the stern, so thrust and moment are as follows,

*τ* = [*τ*_1_, *τ*_3_]′ = [*N*_1_ + *N*_2_, (*N*_1_ − *N*_2_)·*d*/2]′ (4)

where *N*_1_ and *N*_2_ are the thrust of left and right thrusters respectively, and *d* is the distance between the left and right thrusters’ rotated axes that equals to 1 m.

**Figure 2:**
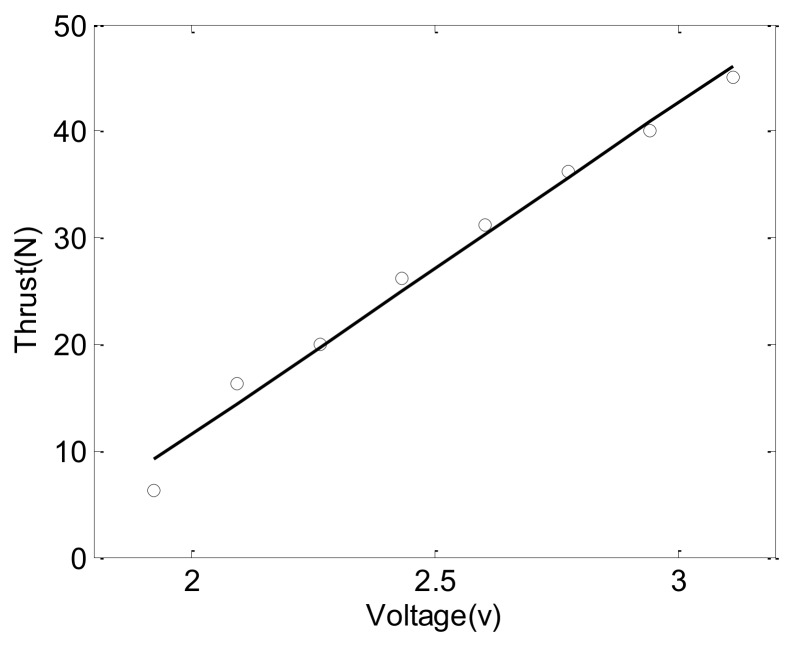
Relationship between control voltage and thrust.

In the USBV’s control system, the revolving speeds of two thrusters are controlled by two brushless DC motor actuators which are regulated by control voltage *v*_i_. The relation between control voltage and thrust is measured by a lake test in 2013, which is given in Figure 2. The circle points are the measurement value, and the line is the linear fitting result. The linear fitting relationship for a thruster between control voltage and thrust is as follows,

(5) $$N_{i}=a\cdot \upsilon_{i}+b=31.1\cdot \upsilon_{i}-50.7,i=1,2$$

## 3. Heading and Velocity Coupled Nonlinear Controller Design

In USBV model Equations (2) and (3), the state variables of USBV are *u*, *v*, *r*, and x, y, *ψ* are output variables, and *v*_i_ are the input variables in Equation (5). Since the sway motion in Equation (3) involved *v* is a non-input, i.e. $\tau_{2}$ = 0, it can be also proved as passive-boundedness and uniformly bounded [27]. Thus, the sway motion is unnecessary, considering the controller design. Here, *α* = [*α*_1_, *α*_2_, *α*_3_] are defined as virtual controls, so new state variables are expressed as

(6) z=x−α

here ***z*** = [*z*_1_, *z*_2_, *z*_3_], ***x*** = [*ψ*, *u*, *r*].

The USBV’s motion model (3) can be transformed as

(7) $$\begin{array}{l} {\dot{z}}_{1}=\dot{\psi }-{\dot{\alpha }}_{1}=r-{\dot{\alpha }}_{1}\\ {\dot{z}}_{2}=\dot{u}-{\dot{\alpha }}_{2}=\frac{m_{22}}{m_{11}}vr-\frac{d_{11}}{m_{11}}u\left|u\right|+\frac{1}{m_{11}}\tau_{1}-{\dot{\alpha }}_{2}\\ {\dot{z}}_{3}=\dot{r}-{\dot{\alpha }}_{3}=\frac{m_{11}-m_{22}}{m_{33}}uv-\frac{d_{33}}{m_{33}}r\left|r\right|+\frac{1}{m_{33}}\tau_{3}-{\dot{\alpha }}_{3} \end{array}$$

Step1 (Design for *τ*_3_): The Lyapunov candidate function is chosen as

(8) $$V_{1}=\frac{1}{2}z_{1}^{2}$$

Thus, differentiating *V*_1_ with respect to time yields

(9) $${\dot{V}}_{1}=z_{1}{\dot{z}}_{1}=z_{1}(r-{\dot{\alpha }}_{1})=z_{1}(z_{3}+\alpha_{3}-{\dot{\alpha }}_{1})$$

The virtual control *α*_3_ is defined as $\alpha_{3}=-k_{1}z_{1}+{\dot{\alpha }}_{1}$, which is substituted into Equation (9), and we have

(10) $${\dot{V}}_{1}=-k_{1}z_{1}^{2}+z_{1}z_{3}$$

Based on Equation (10), the Lyapunov candidate function is chosen as

(11) $$V_{2}=V_{1}+\frac{1}{2}z_{3}^{2}=\frac{1}{2}z_{1}^{2}+\frac{1}{2}z_{3}^{2}$$

Then, differentiating *V*_2_ with respect to time yields

(12) $$\begin{array}{l} {\dot{V}}_{2}=z_{1}{\dot{z}}_{1}+z_{3}{\dot{z}}_{3}\\ =z_{1}(z_{3}+\alpha_{3}-{\dot{\alpha }}_{1})+z_{3}(\frac{m_{11}-m_{22}}{m_{33}}uv-\frac{d_{33}}{m_{33}}r\left|r\right|+\frac{1}{m_{33}}\tau_{3}-{\dot{\alpha }}_{3})\\ =-k_{1}z_{1}^{2}+z_{3}(\frac{m_{11}-m_{22}}{m_{33}}uv-\frac{d_{33}}{m_{33}}r\left|r\right|+\frac{1}{m_{33}}\tau_{3}-{\dot{\alpha }}_{3}+z_{1}) \end{array}$$

Thus, the input controller is defined by

(13) $$\tau_{3}=d_{33}r\left|r\right|-(m_{11}-m_{22})uv+m_{33}({\dot{\alpha }}_{3}-z_{1})-m_{33}k_{3}z_{3}$$

which is substituted into Equation (9), and we can get

$${\dot{V}}_{2}=-k_{1}z_{1}^{2}-k_{3}z_{3}^{2}.$$

Step 2 (Design for *τ*_1_): The Lyapunov function is chosen as

(14) $$V_{3}=V_{2}+\frac{1}{2}z_{2}^{2}=\frac{1}{2}z_{1}^{2}+\frac{1}{2}z_{2}^{2}+\frac{1}{2}z_{3}^{2}\;$$

Then differentiating *V*_3_ with respect to time yields

$${\dot{V}}_{3}={\dot{V}}_{2}+z_{2}{\dot{z}}_{2}={\dot{V}}_{2}+z_{2}(\frac{m_{22}}{m_{11}}vr-\frac{d_{11}}{m_{11}}u\left|u\right|+\frac{1}{m_{11}}\tau_{1}-{\dot{\alpha }}_{2}).$$

Thus, another input controller is defined as

(15) $$\tau_{1}=d_{11}u\left|u\right|-m_{22}vr+m_{11}({\dot{\alpha }}_{2}-k_{2}z_{2})\;$$

then we have

(16) $${\dot{V}}_{3}=-k_{1}z_{1}^{2}-k_{2}z_{2}^{2}-k_{3}z_{3}^{2}\;$$

where *k*_1_, *k*_2_, *k*_3_ are three positive constants.

**Theorem** **1.** *For the USV motion model Equation (7), when α*_1_ *= ψ*_d_ *, α*_2_ *= u*_d_ *,* $\alpha_{3}=-k_{1}z_{1}+{\dot{\alpha }}_{1}$ *,* ${\dot{\alpha }}_{3}=-k_{1}(r-r_{d})+{\dot{r}}_{d}$ *, the asymptotical stability for ψ- ψ*_d_ *, u-u*_d_ *can be achieved by the input control law (13) and (15).*

**Proof.**  Using the control law (13) and (15), USV’s motion model Equation (7) can be transformed into,

(17) $$\left[\begin{array}{c} {\dot{z}}_{1}\\ {\dot{z}}_{2}\\ {\dot{z}}_{3} \end{array}\right]=\left[\begin{array}{ccc} -k_{1} & 0 & 1\\ 0 & -k_{2} & 0\\ -1 & 0 & -k_{3} \end{array}\right]\left[\begin{array}{c} z_{1}\\ z_{2}\\ z_{3} \end{array}\right]\;$$

According to Lyapunov stability’s condition (14) and (16), *z* system is globally asymptotically stable around equilibrium point *z* = 0. Whereas, based on linear relation between control law *τ* and control voltage signal *v*_1,2_ in Equations (4) and (5), we get

(18) $$\begin{array}{l} \upsilon_{1}=(\tau_{1}+2\tau_{3}/d-2b)/2a\\ \upsilon_{2}=(\tau_{1}-2\tau_{3}/d-2b)/2a \end{array}$$

thus, uniqueness of control voltage *v*_1,2_ for the brushless DC motors is assured. □

## 4. Simulation Results

**Figure 3:**
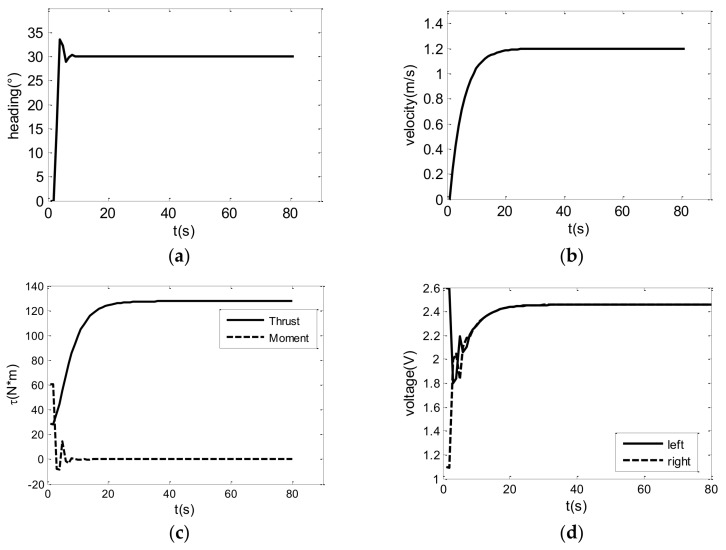
Simulation results of 30° heading control and 1.2 m/s velocity control: (**a**) actual heading of the USV; (**b**) actual velocity of the USV; (**c**) thrust and moment of the propellers; and (**d**) control voltage of the propeller actuators.

In order to verify the proposed algorithm, the heading and velocity control is simulated based on the USV motion model Equation (7), using control law Equation (18). In the simulation, the initial heading and velocity are totally fixed as 0, and the expected heading and velocity are set as 30° and 1.2 m/s, respectively, and the simulation results are shown in Figure 3. In the control law, the parameters *k*_1_, *k*_2_, *k*_3_ are chosen as 0.2, 0.2, 2, respectively, and USBV’s hydrodynamic coefficients in Table 1 are adopted for model Equation (7). It is seen that the expected heading and velocity are followed automatically and successfully in Figure 3a,b. The related thrust and control voltages for propellers aren’t urgent in Figure 3c,d, and saturation also does not appear, which denotes practicability of the proposed algorithm.

## 5. Experiment Results

### 5.1. The USBV

**Figure 4:**
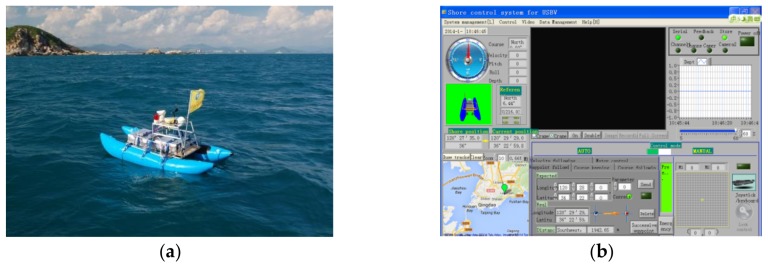
(**a**) Unmanned Surface Bathymetry Vehicle (sea trial around Wuzhizhou Island, 2016); and (**b**) shore control software interface of the USBV.

USBV is based on a catamaran with two propellers in the stern, where the length is 2.8 m and the width is 1.5 m, which is shown in Figure 4a. Based on an embedded microcomputer, a main control system is constructed for sensor processing, control command sending, and autonomous control responding. The integrated sensors contain a DGPS (Trimble DSM232, Sunnyvale, CA, USA), a digital compass (Honeywell 3000, Morris Plains, NJ, USA), a IMU module (MicroStrain 3DM-GX3-25, Williston, FL, USA), a portable echosounder (Hi-Target HD 360, Guangzhou, China), a camera (HIK VISION DS-2DE7230IW, Hangzhou, China) and a weather station (AIRMAR 200WX, Milford, CT, USA) providing temperature, wind speed, and direction. The sensors are integrated in the main control system by RS 232 interface, which measures the state of the USBV synchronously in 1 Hz. For example, the DGPS supplies position and velocity for the USBV, and the compass and IMU module synthetically supply the attitude and angle velocity, and the echosounder measures the depth under the USBV, and the camera can monitor the environment around the USBV. Some data are state variables which are used in the heading and velocity’s control. Two 24 V 400 W propellers are used in the stern propulsion system, which are controlled by two brushless DC motor actuators separately. The ship’s steering is accomplished by differing revolving speed of two propellers. The two 24 V 115 Ah and 12 V 20 Ah batteries are used as driven energy and main control system energy, respectively. In the aspect of waterproof, the main control system and lithium batteries are separated into two stainless steel boxes, and the batteries’ box is in the stern. The shore control unit consists of wireless modules, a battery and a laptop. A GUI (Graphical User Interface) of the shore control software (Figure 4b) programmed by LabVIEW is used to display and store the transmission data, such as depth, position, attitude, control feedback and video, and to send remote and autonomous control commands.

### 5.2. Experiment Results and Analysis

#### 5.2.1. Lake Experiment

**Figure 5:**
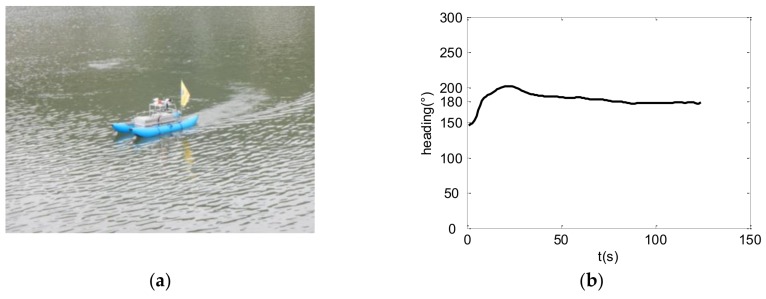

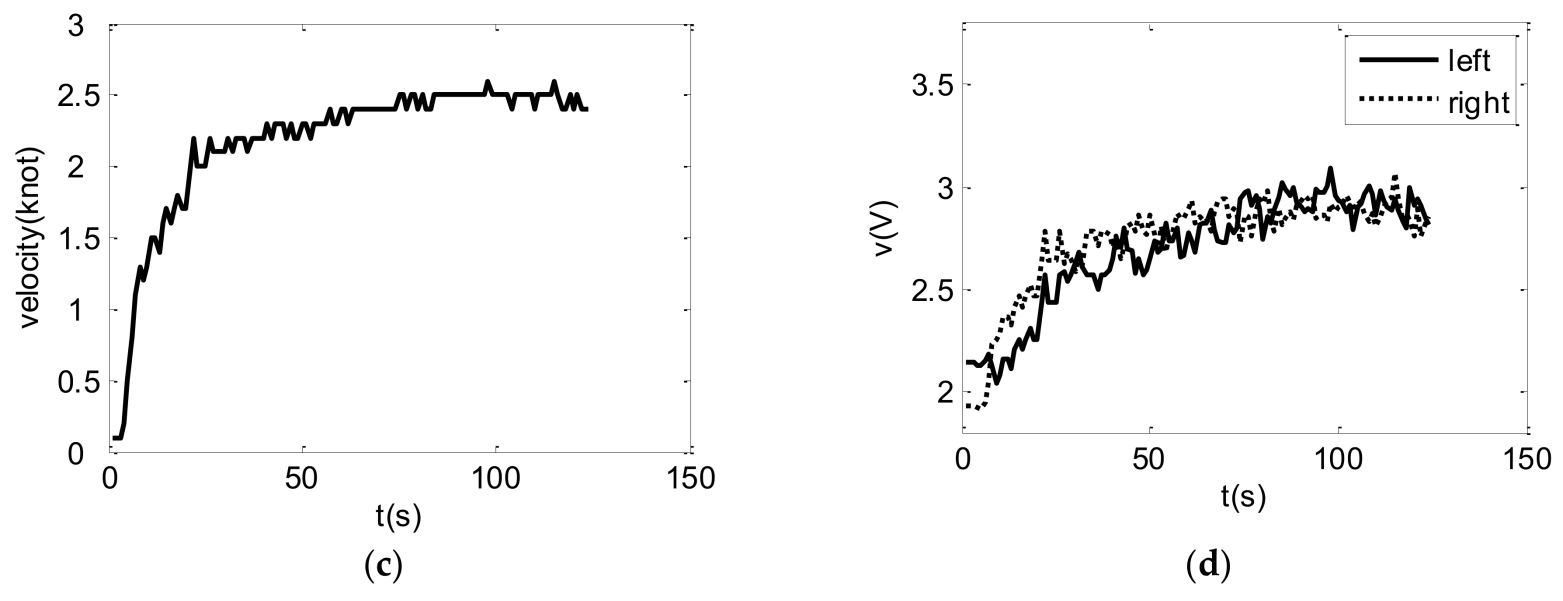
Experiment result for heading and velocity control: (**a**) the USBV in the lake experiment; (**b**) autonomous control for 180° expected heading; (**c**) autonomous control for 2.3 knot expected velocity; and (**d**) the control voltage of left and right propellers.

The control law for the USBV was verificated by experiments at DangLu reservoir in Lianyungang city in China on 7–12 July, 2015 (Figure 5a). In USBV, the Triblem DGPS and the Honeywell 3000 digital compass are used to provide its position, velocity, and heading. The control algorithm is performed by 1 Hz in the USV’s embedded computer. The USBV for the lake experiment is shown in Figure 5a. The experiment results for USBV with 180° expected heading and 2.3 knot expected velocity are given, respectively, in Figure 5b,c, and the corresponding control voltages of left and right propellers are shown in Figure 5d. The environment wind is measured using the weather station integrated in USBV. The maximum velocity of wind is approximately 3.5 m/s, and the direction is inconstant especially in the beginning. Under the wind condition, the expected heading and velocity are successfully tracked at the same time. In Figure 5b, the USBV’s heading converges to 180° expected heading in about 30 s, and the tracking is very stable. In Figure 5c, 2.3 knot expected velocity be also achieved in about 30 s, and the actual velocity shows sawtooth due to the measure precision with 0.1 knot in velocity of the DGPS. It is shown that the heading and velocity are controlled simultaneously by regulating the control voltages of the two thrusters in Figure 5d.

#### 5.2.2. Sea Experiment

**Figure 6:**
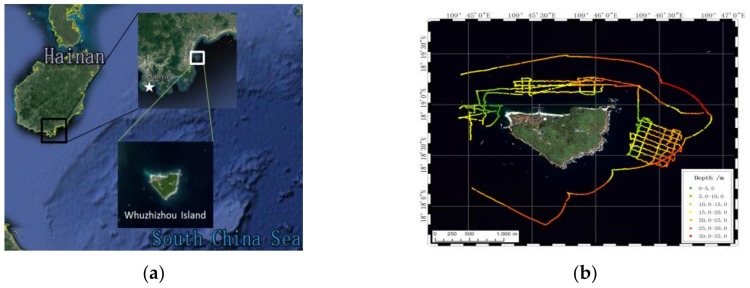
(**a**) Location of the sea experiment around Wuzhizhou Island, Sanya City, China; and (**b**) USBV’s trajectories with depth measurement.

Application experiment for the USBV depth measurement was performed around Wuzhizhou Island on 3–6 January, 2016 (Figure 4a) [28]. Wuzhizhou Island is located at 18°18′40″ N, 109°45′45″ E, off the Haitang Bay, Sanya City, Hainan Province, China, which is 2.7 km offshore from the Haitang Bay (Figure 6a). During 4 days of experiments, the USBV was used for a total time and distance of approximately 16 h and 55 km, respectively. The USBV’s trajectories with instantaneous depth (denoted by a colored line) are shown in Figure 6b, where the largest successive time and distance for measurement were approximately 6 h and 20 km on the last day, respectively. In Figure 6b, the USBV’s trajectories contains “lawn mower” curves and lines around Wuzhizhou Island. In the sea experiment, the USBV’s autonomous system is verificated below the third level of the sea stage.

## 6. Conclusions

In this paper, a heading and velocity coupled nonlinear controller for USV is designed and verificated. In USBV’s modelling, the relationships between drag terms and velocity are quadratic, which are shown in the tow experiment for USBV. Based on a quadratic identified drag terms, USBV’s nonlinear motion model is accomplished. Using backstepping technology, a nonlinear controller is acquired for USV in surge and yaw direction based on Lyapunov function. The efficacy of the USBV and its autonomous is also validated by lake and sea experiments, and the heading and velocity are controlled simultaneously. In the future, some controllers for USV considering wind, wave and current disturbances will be designed and tested, which will improve the robustness of autonomous control systems of the USBV.

## Figures and Tables

**Table 1:** USBV’s hydrodynamic coefficients.

Coefficients	Values
*d*_11_/(kg/m)	88.6
*d*_22_/(kg/m)	181.6
*d*_33_/(kg·m/rad^2^)	687.4
*m/*kg	100.0
$X_{\dot{u}}$/kg	−20.0
$Y_{\dot{v}}$/kg	−21.6
$N_{\dot{r}}$/(kg·m/rad)	−206.2
*I*/(kg/m)	20.0
